# MacMillan & Co.

**Published:** 1896-07

**Authors:** 


MacMillan & Company, 66 Fifth Avenue, New York, have issued a handsome volume entitled *The Fundus Oculi*, with an ophthalmoscopic atlas illustrating its physiological and pathological conditions, by W. Adams Frost, F. R. C. S. It is a quarto volume, extra cloth, gilt top, price, $18.00. The same firm have also in press *A System of Medicine* by many writers, edited by Thomas Clifford Allbut, M. A., M. D. etc., to be complete in five volumes, medium octavo. Price, cloth, $5.00 net.

